# The dissemination and implementation of trauma-focused cognitive behavioural therapy for children and adolescents in seven European countries

**DOI:** 10.1186/s12913-024-11689-3

**Published:** 2024-10-08

**Authors:** Elisa Pfeiffer, Johanna Unterhitzenberger, Pia Enderby, Aino Juusola, Zlatina Kostova, Ramon J. L. Lindauer, Sanna-Kaija Nuotio, Poa Samuelberg, Tine K. Jensen

**Affiliations:** 1 Department of Child and Adolescent Psychiatry/Psychotherapy, Ulm University, Steinhoevelstraße 1, 89075 Ulm, Germany; 2 Faculty of Social Sciences, Rosenheim Technical University of Applied Sciences, Hochschulstrasse 1, 83024 Rosenheim, Germany; 3 Department of Biomedical and Clinical Sciences, Linköping University, National Center on Knowledge on Violence against Children, Barnafrid, Linköping, 581 83 Sweden; 4 Finnish Institute for Health and Welfare, Mannerheimintie 166, 00100 Helsinki, Finnland; 5 Åbo Akademi University, Tuomiokirkontori 3, Turku, 20500 Finnland; 6 Department of Psychiatry, University of Massachusetts Chan Medical School, 55 Lake Avenue North, Worcester, 01655 United States of America; 7 Department of Child and Adolescent Psychiatry and Levvel, Academic Centre for Child and Adolescent Psychiatry, Amsterdam UMC, location AMC, University of Amsterdam, Meiberdreef 5, Amsterdam, 1105AZ the Netherlands; 8 Department of Forensic Unit for Children and Adolescents, Turku University Hospital, Yliopistokatu 26 B, Turku, 20100 Finland; 9 Department of Psychology, University of Oslo, Forskningsveien 3A, Oslo, 0373 Norway; 10 Norwegian Centre for Violence and Traumatic Stress Studies, Gullhaugveien 1, Oslo, 0484 Norway

**Keywords:** Children and adolescents, TF-CBT, Dissemination, Implementation, Europe

## Abstract

**Background:**

There is broad scientific evidence for the effectiveness of individual trauma-focused evidence-based treatments (EBTs) such as “trauma-focused cognitive behavioural therapy” (TF-CBT) for children and adolescents with posttraumatic stress symptoms. However, there is a significant research-to-practice gap resulting in traumatized children in high-income countries in Europe having only very limited access to these treatments. The aim of this study was, therefore, to identify common barriers and successful dissemination and implementation (D&I) strategies of evidence-based trauma-focused treatments (in particular TF-CBT) in seven European countries.

**Methods:**

For this study, we chose a mixed-method approach: an online survey among certified European TF-CBT trainers (*N* = 22) and the collection of country-based narratives from TF-CBT experts in different European countries (Finland, Germany, Italy, Netherlands, Norway, Sweden).

**Results:**

Common modifiable barriers to the implementation of TF-CBT were identified on different levels (e.g. government or treatment level), and successful D&I strategies were highlighted across all countries, such as translations of materials. Additionally, the experts from the country narratives put together a broad overview of TF-CBT research in Europe.

**Conclusions:**

The results of this study revealed that especially learning collaborations and the development of joint European efforts in funding and researching D&I strategies are crucial for future implementation of trauma-focused EBTs in Europe.

**Supplementary Information:**

The online version contains supplementary material available at 10.1186/s12913-024-11689-3.

## Introduction

Children and adolescents have a high risk of experiencing traumatic events [27]. The conditional prevalence for post-traumatic stress disorder (PTSD) after a traumatic event is estimated to be about 16% [3]. Over the last two decades, several psychotherapeutic treatment models for children and youth with post-traumatic stress disorder (PTSD) have been developed and have proven to be effective in reducing not only PTSD but also other trauma-related disorders. The strongest support is for trauma focused cognitive behavioural treatment (CBT) based approaches such as “trauma-focused cognitive behavioural therapy” (TF-CBT; [8]). In addition to individual treatments, group-based programs such as Cognitive Behavioural Intervention for Trauma in Schools (CBITS; [50]) or “Mein Weg” [35] have garnered evidence about reducing post-traumatic sequelae.

Despite the numerous trials on trauma-focused PTSD treatments [30, 47] for children and adolescents in high- and low-income countries around the world [4], as well as a growing number of studies on the change mechanisms [21, 22, 34] of these treatments, there is a significant research-to-practice gap and insufficient knowledge about successful implementation strategies to promote the use of evidence-based treatments (EBTs) among practitioners [24]. Kratz et al. [24] describe that the findings from efficacy studies on the treatment of youth with PTSD do not find their way to routine mental health care. This prevents traumatized youth from receiving the most promising treatments and therapists from providing treatments that are effective for most children and adolescents with PTSD [46]. Previously identified common (modifiable) barriers to the implementation of EBTs in mental health services can be categorized in five levels: (1) Patient level (e.g. lack of motivation, no accurate diagnosis); (2) therapist level (e.g. distorted beliefs on EBTs or alternative preferences); (3) treatment level (e.g. difficulty in identifying and choosing from EBTs); (4) organization level (e.g. lack of administrative support and training, scepticism about EBTs); and (5) government level (e.g. health care structure and policy; lack of providers trained in EBTs) [19]. There is an increasing number of relevant conceptual frameworks for dissemination and implementation (D&I) research which serve multiple purposes such as identifying the indicators and outcomes of implementation success. In this manuscript we define implementation as “(…) the process of putting to use or integrating evidence-based interventions within a setting“ ([33], p.1) and dissemination as “(…) the active approach of spreading evidence-based interventions to the target audience via determined channels using planned strategies.” ([38], pp.117–123).

There is a growing body of research on large-scale efforts such as nationwide programs to disseminate and implement EBTs, in particular TF-CBT, in the USA. A recent systematic review by Powell et al. [37] of determinants of implementing evidence-based trauma-focused interventions for children and adolescents encompassed a total of 23 articles on TF-CBT and CBITS. Only four studies reported on the implementation of trauma-informed care outside the USA (Zambia, Kenya, Tanzania) – none of them in Europe.

Although there are large efficacy trials on TF-CBT in Norway [21], Netherlands [11] and Germany [17], only sparse information is available on the successful broad implementation models in European care systems. One example for the implementation of TF-CBT into a European care system is the project “TF-CBT Ukraine” [36] in which the model is implemented as a large training program for Ukrainian therapists during war time. Two other large implementation efforts are made in Germany with the projects “BEST FOR CAN” [39] for children who report abuse and “BETTERCARE” [40] for refugee children. Lastly, in Norway there is an ongoing national implementation program currently being evaluated [13]. Exploring these broader implementation models is especially challenging as mental health care for youth is organized differently across Europe. In addition, it is almost impossible to compare access to EBTs for PTSD for traumatized children and youth across Europe. This study aimed to shed some light on current TF-CBT D&I efforts in several European countries by exploring the unique perspectives of international TF-CBT trainers in their efforts to disseminate this model in their respective European countries. This research builds on a recent report on trauma care in Europe [42] which focused on a general description of the current state of care for survivors of trauma in 15 European countries. This particular study adds to extant literature by offering clarity around trauma-focused care for children and adolescents from a child trauma expert perspective. This study could, therefore, serve to inform the field of D&I research on joint barriers but also on solutions for the D&I of EBTs for traumatized children and adolescents in Europe. It can likewise promote the effective integration of EBTs into health care systems. For this study, we chose a mixed-method approach: an online survey among certified European TF-CBT trainers (who had completed the TF-CBT train-the-trainer (TTT) program run by the treatment developers) as a quantitative and qualitative approach and the collection of country-based narratives from TF-CBT experts as an additional qualitative approach. In this context, the country narratives provided a more thorough description of each country’s specific D&I context.

## Methods

### Online survey

#### Sample

The sample consisted of certified TF-CBT trainers in Europe who had been trained by the treatment developers. All trainers working in Europe were invited to participate in the survey, independently of their current occupation and when they underwent TF-CBT training.

#### Study design

The trainers were invited to participate in the survey via an email invitation and announcements in regular trainer meetings. The Institutional Review Board at Ulm University approved this study. All participants gave their written informed consent. Study authors conducted the country narratives and could also have been study participants in the survey (data is anonymized). The survey was conducted between June and August 2021 and the country narratives were written by the individual authors between May and August 2022.

#### Measures

Since there are no standardized measures for assessing D&I efforts in European mental health care systems, we used non-standardized and self-developed questions (see appendix 1). We included single- and multiple-choice questions as well as open questions. The answer options were reviewed by four trainers from four countries to make sure they covered every mental health system in an optimum manner.

#### Statistical analyses

The single and multiple choice questions were descriptively analyzed via IBM SPSS Statistics Version 26. In case of any discrepancies between participants of the same countries we used mean scores of the answers (e.g. number of trained clinicians). The open questions in the survey were analyzed according to Mayring’s qualitative content analysis [29]. We determined categories in a structured manner, which were then analyzed quantitatively by number of namings. EP and JU analyzed qualitative responses; in case of discrepancies, ZK was consulted. As the answers were very short or only bullet points, there were no discrepancies found. The above mentioned framework by Harvey and Gumport [19] was used in a slightly modified version to structure the results.

### Country narratives

For writing the short narratives, we identified TF-CBT experts from all European countries represented in the online survey, based on their contribution to their field. They were invited to write short narratives based on the instruction in appendix 2.

Furthermore, all experts were invited to summarize their TF-CBT related research in an online supplement (see appendix 3). Experts were asked to submit their narratives between May and August 2022. Only one expert (UK) failed to comply with the request. After submission, the lead authors (JU, ZK and EP) reviewed all narratives independently, edited the narratives for clarity and consistency, shortened them and made sure they followed the same structure. All narratives were organised by the lead authors into three sections: (1) mental health services for traumatized children; (2) TF-CBT implementation; (3) research on TF-CBT.

## Results

### Results of the online survey

#### Sample description

Altogether *N* = 22 certified trainers in Europe whose contact details were available, were invited to complete the survey and *n* = 17 trainers representing seven different countries (Germany *n* = 5; Netherlands *n* = 3; Sweden *n* = 3; Finland *n* = 2; Norway *n* = 2; UK *n* = 1; Italy *n* = 1) participated. In terms of their TF-CBT experience, the participants had treated M = 44.97 (SD = 51.58; range = 10–200) patients with TF-CBT themselves, *n* = 16 (94.1%) had implemented TF-CBT training programs and case consultations and *n* = 5 (29.4%) had also conducted research on TF-CBT.

#### Implementation of TF-CBT

The common settings/institutions for TF-CBT implementation were mostly mental health clinics – outpatient setting (*n* = 7; 100%) and private practice (*n* = 5; 71%) (Table 1). Current numbers of TF-CBT treatment providers and TF-CBT trainers in all countries are given in Table 2.

**Table 1:** Common settings /institutions for TF-CBT in each country

	Country ^a^
Mental health clinics – inpatient setting	Germany, Italy, Netherlands
Mental health clinics – outpatient setting	Sweden, Finland, Germany, Italy, Netherlands, Norway, UK
General hospitals	Netherlands
Psychotherapy training institutes	Finland, Germany, Italy, Netherlands, Sweden
Schools	Finland
Child welfare programs	Netherlands
Juvenile justice system	Finland, Netherlands
Private practice	Finland, Germany, Netherlands, Sweden, UK
Other: voluntary sector	UK

Note. Indicated per country (*n* = 7 countries) ^a^at least one person from the respective country indicated yes

**Table 2:** Overview of numbers of treatment providers in 2021

	Finland	Germany	Italy	Netherlands	Norway	Sweden	UK^b^
Number of TF-CBT trained clinicians altogether	120	400–600	0^a^	100–300	600	10–400	100
Number of TF-CBT trained clinicians each year	40	100	20	75–100	100	100	60
Number of clinicians delivering TF-CBT	60	150	20	200	377	22	15
Number of trainers certified by the treatment developers	2	14	1	4–5	12	9–10	1

Note. These questions were answered by one trainer for each country. Trainers were asked to provide approximate numbers as it is very difficult to have an exact number

^a^TF-CBT training programs started in late 2021, prior to the survey

^b^The trainers indicated that the numbers only included people they had personally trained

The most prevalent implementation strategies include the development of training programs/ opportunities, the development of an evidence-based engagement strategy (i.e., support or incentives for providing EBTs) and materials in the country’s language. An additional strategy was follow-up visits with trained clinics and leadership programs (see Table 4 together with dissemination strategies).

##### TF-CBT training

Only three out of seven countries have a certification program for TF-CBT therapists^1^. The prerequisites for the participation of clinicians in a TF-CBT training program varied considerably from country to country. In terms of the education level, in most countries the clinicians need to have a master’s degree and have to be involved in a clinical training program after completing their university degree (Table 3). Specific TF-CBT related prerequisites prior to implementing TF-CBT with patients include the TF-CBT web-training (5 countries), reading the TF-CBT manual (5 countries), participation in a 2–3 day basic TF-CBT workshop (7 countries) and case consultation (5 countries) (Table 3).

**Table 3:** Training requirements for a person wishing to participate in TF-CBT training (*N* = 7 countries)

**Requirements**	**n(countries)**	**Countries**
High school diploma	0	
Bachelor’s degree	1	Sweden
Master’s degree	5	Finland, Germany, Italy, Netherlands, Sweden
Clinical training as part of a university degree (bachelor’s)	1	Finland
Clinical training as part of a university degree (master’s)	2	Finland, Norway
Clinical training program after university degree	6	Finland, Germany, Italy, Netherlands, Norway, UK
Part of vocational training /after vocational training	0	
Certain specializations	1	Norway
Other	1	UK: Doctoral level clinical psychology trainees; generally have a mental health qualification
*TF-CBT-related prerequisites for the delivery of TF-CBT as a therapist/clinician in each country*		
**TF-CBT-related prerequisites**	**n (countries)**	**Countries**
TF-CBT web training	5	Finland, Germany, Netherlands, Norway, Sweden
Read TF-CBT manual	5	Finland, Germany, Netherlands, Norway, Sweden
Participate in 2–3 day basic training	6	Finland, Germany, Italy, Netherlands, Norway, Sweden
Case consultation	5	Finland, Italy, Netherlands, Norway, Sweden
Specific number of training cases under supervision	6	Finland, Italy, Netherlands, Norway, Sweden, Italy
Other:	3	Germany & Italy: there are no nationwide requirements yet Norway: Follow-up course after one completed case

##### Training programs

The TF-CBT training is mostly delivered by TF-CBT trainers certified by the developers (*n* = 7 countries) and TF-CBT therapists (*n* = 3 countries). In three countries TF-CBT training is also run by clinicians with no specific TF-CBT training, as there is no certification program. Additionally, respondents from *n* = 2 countries indicated that private organizations and national centres offered TF-CBT training in collaboration with a university.

##### Case consultations

TF-CBT training is normally accompanied by specific TF-CBT case consultation in all participating countries. However, several participants indicated in an open question that trained participants do not always have access to the case consultation afterwards due to organizational reasons or because of a lack of time or funding. In all countries, case consultations are delivered by TF-CBT trainers. In *n* = 3 countries, case consultations were also delivered by TF-CBT therapists and in *n* = 1 country by other clinicians. Approximately 5 (M = 5.37; SD = 2.83; range 2.5–13.5) clinicians participate in one case consultation group with one trainer.

#### Dissemination strategies

Three dissemination strategies were reported pertaining to publications and learning collaboratives (see Table 4). In an open question, one participant also mentioned that they had developed a TF-CBT homepage.

**Table 4:** Current implementation strategies per country

	*n*	Countries^a^
Development of training institutes which offer TF-CBT training	5	Finland, Germany, Netherlands, Norway, Sweden
Cooperation with institutes in order to implement TF-CBT	7	Finland, Germany, Italy, Netherlands, Norway, Sweden, UK
Development of an evidence-based engagement strategy	3	Netherlands, Norway, UK
Development of an implementation manual	2	Netherlands, Sweden
Development of own TTT-program	0	None
Development of toolbox or workbook in your language	6	Finland, Germany, Italy, Netherlands, Norway, Sweden
Development of TF-CBT web training	2	Germany, Norway
*Current Dissemination strategies per country*		
Development of regional learning collaboratives for agencies / institutions / centres	1	Finland
Manual publication in respective language	6	Finland, Germany, Italy, Netherlands, Norway, Sweden
Publication of scientific studies on TF-CBT	4	Germany, Netherlands, Norway, Sweden

^a^at least one person from the respective country answered yes

#### Barriers and requirements

The perceived barriers reported by participants to the current implementation and future dissemination of TF-CBT were categorized in five levels: government level, organization level, therapist level, treatment level (treatment meaning TF-CBT in particular) and trauma-related level (Table 5). Most barriers were on the treatment level (e.g. lack of trainers or materials), but also on the government level (e.g. lack of funding). Most barriers were reported by individual countries instead of the entire group, which might reflect that perceived barriers are quite country-specific and need to be addressed in each country differently. The necessary requirements for larger scale implementation and dissemination of TF-CBT partly reflected the barriers (e.g. lack of TF-CBT trainers) but also highlighted the need for more country-based implementation strategies (e.g. certification programs) (Table 5). A more general need for routine trauma screening and assessment, as well as treatment evaluation, has been set out in barriers and requirements.

**Table 5:** Barriers and necessary requirements for the dissemination of TF-CBT in Europe

	Barriers for the implementation and dissemination of TF-CBT	Necessary requirements for wider scale implementation and dissemination of TF-CBT
Government level	Lack of funding (1) No obligation to implement evidence-based treatments (EBTs) (1)	Funding organization (2) More focus on EBTs (1)
Organization level	Insufficient support from leaders (4) High staff turnover rates (2) Lack of cooperation between different clinics/ facilities (1)	More engagement of leaders (e.g. responsibility of routine trauma screening and continuous training in the case of staff turnover) (1)
Therapist level	Therapists do not start treatment after training (2) High workload of treatment providers (1)	More knowledge (e.g. on the advantages of TF-CBT compared to other trauma-focused EBTs) (2) More trained treatment providers (1)
Treatment level	Lack of TF-CBT trainers (3) Not enough translated materials (1) TF-CBT is not well-known (1) TF-CBT is less popular than other (trauma-focused) treatments (3) Lack of an institution which specifically organizes TF-CBT training/case consultation (1) Lack of TF-CBT training/case consultation opportunities (2)	More trainers (3) Translation of web training (1) More translated materials (1) Development of a country-based TF-CBT association (1) More flexibility in extending the program (e.g. into a more intensive treatment plan) (2) More focus on systemic/family participation (1) More guidance from the developers (1) Development of evidence-based implementation strategies (2) European Train-the-Trainer Program (1) Certification and licensing programs (2)
Trauma-related level	Not enough sensitivity to trauma (1) Trauma-related myths (1) Lack of screening for trauma/ PTSD (2)	More guidance in choosing and implementing assessment tools (1) Implementation of routine trauma screening and treatment evaluation (3)

#### Presentation of country narratives

##### Finland

1) Mental health services for traumatized children: In Finland, the mental health system for children and adolescents is public. Since 2023, Finland was divided into 23 counties in charge of offering health and welfare services. Even though the availability of mental health services differs among counties or regions, the Finnish mental health system is founded on the principle of equal accessibility.

Screening for PTSD varies throughout the country and the implementation of routine trauma screening as well as evidence-based trauma treatments in the mental health systems is underway. A variety of PTSD questionnaires are in use but none of them has been validated in a Finnish population. The national Barnahus project launched a national survey about the availability of different trauma-focused models in 2020 [26]. The objective of the Barnahus project is to mainstream practices compliant with the Barnahus standards in investigation processes of suspected cases of violence against children as well as to provide support and treatment for children who have encountered violence. The project is funded by the Ministry of Social Affairs and Health, coordinated by the Finnish Institute for Health and Welfare. According to the survey, a wide variety of treatment models are on offer, but not all of them are evidence-based as suggested by the national guidelines (2020): they are TF-CBT, Eye Movement Desensitization and Reprocessing (EMDR; [45]), Narrative Exposure Therapy (NET; kidNET [43, 44] and other CBT-based trauma treatments. The availability of these treatment models has mainly been concentrated in the southern and western parts of Finland around the largest cities. During the Barnahus project, access to TF-CBT improved. The public sector is responsible for the equal distribution and implementation of evidence-based trauma treatments. The role of the Finnish University Hospitals is fundamental although the quality, quantity and other aspects of the implementation process need to be monitored at a national level as well.

2) TF-CBT implementation: The TF-CBT pilot started in 2012 with four training programs in 2012–2019 (funded by the Helsinki University Hospital). In 2018, Finland obtained its first two licensed TF-CBT trainers. Almost 80 new TF-CBT therapists underwent training in 2020–2022. Five national TF-CBT supervisors will undergo training as part of the implementation process in 2022-23, together with Linköping University, Barnafrid. In addition, five national coordinators (facilitators) were trained in 2022. These training programs are funded by the Barnahus project. Future funding of the training programs is set to be the future dilemma. The Finnish implementation of TF-CBT has been influenced by the Nordic network of TF-CBT trainers.

3) Research on TF-CBT: So far, no research on TF-CBT has been published in Finland. A future topic could be the suitability of TF-CBT for telehealth therapy, which would be justified by the long distances in Finland.

##### Germany

1) Mental health services for traumatized children: Children and adolescents can access trauma-focused treatments when they enter routine care (public health insurance covers the costs for up to 24 outpatient psychotherapy sessions). However, while every licensed psychotherapist is allowed to treat PTSD, they do not all offer (evidence-based) trauma-focused treatments. The limited availability of treatment options is the main barrier to trauma-focused treatments in Germany. The main trauma treatments available to children and adolescents in Germany are EMDR for children [20], TF-CBT, kidNET, trauma-centred play therapy, psychodynamic imaginative trauma therapy for children (PITT-KID; [25]) and multidimensional psychodynamic trauma therapy for children (MPTT-KJ; [12]. They are based on the curriculum for trauma treatment of the German-speaking Association for Psychotraumatology [10]. A representative study suggests that about 40% of psychotherapists (for both adults and children/adolescents) have undergone specific training in the treatment of trauma. However, it is still unclear whether these were EBTs. There are no special reimbursements or incentives for psychotherapists to use EBTs. Instead, they may practice in line with their best knowledge. The number of treatment facilities that offer EBTs is, therefore, unknown. Although there are validated paediatric PTSD measures in German, the screening for traumatic events and trauma related symptoms in Germany varies considerably. There is no mandatory routine trauma screening in clinics or practices. It is up to the facility and the professional when and if to ask about trauma.

2) TF-CBT implementation: In Germany TF-CBT began with the randomized controlled trial (RCT) “treat child trauma” led by Lutz Goldbeck and supported by Rita Rosner. This resulted in the training of 26 therapists and three trainers. From this cohort of therapists, another 5 therapists were trained as trainers in 2017/18. In 2020 an additional 5 trainers underwent training. In 2018, the German version of the TF-CBT online training (https://tfkvt.ku.de/) was launched. Since then, more than 7000 users registered and 2160 finished the training (16.12.22). As of this publication there is no TF-CBT certification program in Germany which makes it almost impossible to estimate the number of TF-CBT therapists in the country.

3) Research on TF-CBT: In the context of the RCT, several studies on the following topics were published: complex PTSD, treatment alliance and expectancy, therapist effect, non-responder characteristics, grief, change in cognitions as mediator and unaccompanied refugee minors (URMs) (see supplement). In addition, a pilot study on TF-CBT with URMs and a study on cultural applications were published, as well as findings on a group intervention for URMs based on TF-CBT. Recent research focused on D&I in a stepped-care approach for URMs (BETTER CARE) and on the implementation of TF-CBT for children and adolescents after child abuse and neglect (BESTFORCAN).

##### Italy

1) Mental health services for traumatized children: The health system in Italy is public and everyone has free access to a broad range of health services, including mental health services. Childhood trauma falls mainly under the umbrella of the social services system. Referrals of cases of childhood trauma are usually managed by social services (“Consultori Famigliari”) that are part of the municipalities of each town. The referrals are then sent to associations, cooperatives (cooperative), day centres and community homes that mainly offer recreational activities, educational interventions along with school and social support. Other referrals are sent to outpatient neuropsychiatric clinics, where children are mainly assessed for neurodevelopmental or mental health conditions and provided with some short-term stabilization treatment. As a result, there is much less use of evidence-based clinical interventions for trauma.

For trauma-focused treatments, there are no specific protocols or standards of EBTs. Some of the most frequently used treatments for trauma are EMDR, NET and Schema Therapy [54]. The dissemination of EBTs continues to be a challenge for Italian clinicians, as does the acquisition of a systemic vision of care for traumatized youth with shared policies and protocols.

Trauma screening is not routinely included in the procedures of numerous mental health services in Italy. Many clinicians do not utilize standard, evidence-based screening tools to assess trauma exposure or post-traumatic stress in children and youth and most of the therapists who implement trauma screening are clinicians who are specifically trained in trauma-informed interventions.

2) TF-CBT implementation: In Italy TF-CBT training was first carried out in 2021–2022 through the “Italian Society of Cognitive Behavioural Therapy”. The first Italian cohort consisted of 20 clinicians who were either licensed psychotherapists or psychotherapists in training. The second cohort in 2023–2024 consisted of 14 psychotherapists. Future dissemination efforts in Italy should identify strategies that facilitate the cohesive implementation of EBTs, including methods for highlighting TF-CBT as the leading EBT for traumatized youth.

3) Research on TF-CBT: Unfortunately, no research on TF-CBT has been published to date in Italy.

##### Netherlands

1) Mental health services for traumatized children: Since 2015, youth mental health care has been decentralized and funding is channelled through the local municipalities. This means that each mental youth care institution reaches an agreement per municipality as to which care will be purchased. In principle, trauma treatment for children and adolescents is included in the care package.

In recent years, knowledge in the field of psychological trauma in children and adolescents in the Netherlands has been increasingly disseminated, as evidenced by the number of trainings, translated books, symposia that are being organized and guidelines that are being developed. Knowledge of psychological trauma is also increasingly reaching special education schools [32] and specific target groups, such as children with intellectual disabilities [53]. Knowledge in this area is disseminated by national knowledge centres such as the Knowledge Centre for Child and Adolescent Psychiatry [23] and the Netherlands Youth Institute [31].

In the Netherlands, many therapists have been trained in EMDR and there is also an active EMDR association [14]. In addition, therapists have been trained in CBT and specifically in TF-CBT [51]. The main trauma treatments in the Netherlands are TF-CBT and EMDR. These treatments are also included in various guidelines. There are various training and education options for TF-CBT and EMDR.

A national Dutch guideline ‘Signaleren traumaproblemen’ (‘Identifying trauma problems’) was recently developed (https://guidelinesjeugdhulp.nl/trauma/). It recommends several screening tools for trauma and PTSD, as well as the clinical interview “clinician-administered PTSD scale -child and adolescent version” (CAPS-CA; [52]) for specialist trauma diagnostics. The implementation of these trauma screeners and clinical interviews in clinical practice still require the necessary attention.

2) TF-CBT implementation: In 2006, Dutch trauma therapists went to the TF-CBT developers in Pittsburgh, Pennsylvania, USA, to receive training by them in TF-CBT. In the Netherlands there are five trainers/supervisors and three supervisors in TF-CBT. The trainers teach at psychotherapy training institutes that are known throughout the country which means that they have a large reach. In recent years, hundreds of therapists have been trained in TF-CBT. TF-CBT workbooks have been compiled in Dutch for children, adolescents, parents and therapists [5–7], and an online module has been developed.

3) Research on TF-CBT: The Research Department of Child and Adolescent Psychiatry at Amsterdam UMC and Levvel conducted an RCT studying the effects of TF-CBT and EMDR. A second RCT is in the final phase. In addition, biological research has been conducted into predictors of treatment success.

##### Norway

1) Mental health services for traumatized children: The health care system in Norway is public and free of charge. Children are referred to specialized mental health services by their doctor, child protection services or municipal first-line services. TF-CBT is the most widely implemented trauma model for children and adolescents in Norway, with EMDR also available on a limited basis. Trauma screening is mandatory for clinics who are part of the TF-CBT implementation program, and CATS-2 is recommended as the screening tool.

2) TF-CBT implementation: TF-CBT has been implemented in Norway since 2012 and over 80% of the mental health outpatient clinics for children and adolescents have implemented TF-CBT. The training and implementation efforts are financed by the Norwegian Ministry of Health and are run by the Norwegian Centre for Violence and Traumatic Stress Studies (NKVTS).

The ongoing national implementation program was initiated after NKVTS conducted a RCT between 2008 and 2011. In the implementation research program different single implementation strategies were used simultaneously and followed the framework from Core Implementation Components [15] and Exploration, Preparation, Implementation and Sustainment (EPIS; [1]. In the first implementation period (2012–2017), all therapists at the clinics underwent in-person training in trauma and PTSS assessment. A sub-group of therapists received training in TF-CBT. In the second implementation period (2017–2022) the leadership component was strengthened, and the leaders attended a one-year Leadership and Organizational Change for Implementation program to support and strengthen both the implementation leadership and the implementation climate [2].

Our evaluation of the program showed that clinicians and leaders were positive about using TF-CBT, and clinicians found the model helpful and easy to learn. Also, therapists with training in TF-CBT presented fewer symptoms of burnout and higher compassion satisfaction than those with no training. However, clinician turnover and high caseloads were major barriers to implementation. Supervision and leadership support were important for outcome and sustainability. High-intensity therapist training and case consultation seemed to be a key to preventing patient non-response and drop-out. Leadership training seemed to positively affect non-response and drop-out, although it was not enough to compensate for less intensive therapist training.

3) Research on TF-CBT: Several research projects have been conducted on TF-CBT in Norway focussing on the therapeutic alliance, therapy process, parental involvement, outcome trajectories, health economics and implementation strategies (see supplement).

##### Sweden

1) Mental health services for traumatized children: Swedish public healthcare is organized and run by county councils, local authorities or municipalities, and there are both public and private providers. Mental health care for children and youth is free of charge. No trauma-informed care is implemented at an organizational level [42]. Access to EBTs differs greatly from region to region in Sweden. The treatments offered for trauma-related syndromes in child and adolescent psychiatry include TF-CBT, EMDR, Prolonged Exposure -Adolescents (PE-A; [16]), KidNET, Child-parent Psychotherapy (CPP; [28] and trauma-centred play therapy [18]. During recent decades, child and adolescent psychiatry has faced problems caused by large staff turnover and increasing waiting times for children and youth in need of mental health interventions and EBTs [41, 49]. The health system, has established guidelines for screening for violence and abuse [48, 49]. However, there is no national systematized monitoring or systematic follow-up of screening and interventions regarding trauma and trauma-related symptoms within child and adolescent psychiatry. The routines differ broadly between units and professionals.

2) TF-CBT implementation: TF-CBT was introduced in Sweden in 2005. A total of ten licensed Swedish trainers participated in the European TTT program in 2013, 2017 and 2021. The TF-CBT training is conducted at Barnafrid (i.e., the national knowledge centre on violence against children at Linköping University), Save the Children Sweden and in private settings. The lack of a certification program in Sweden makes it more difficult to obtain an estimate of the current number of trained TF-CBT therapists.

Over the last five years, Barnafrid has played a unifying leadership role in the D&I of TF-CBT in Sweden. Various initiatives have been taken to support and maintain fidelity and skills in TF-CBT – some in collaboration with Norway and Finland. They include enhancing infrastructure (National, Nordic and International TTT networks), therapist resources (a Swedish translation of the TF-CBT manual [9], TF-CBT Toolbox in press, TF-CBT workbooks for children and caregivers translated into Danish, Finnish, Norwegian, Spanish, and in press in English and Ukrainian) and education (Digital TF-CBT basic training, Supervision Training in TF-CBT – a pilot program in collaboration with Finland).

The limited child and adolescent psychiatry capacity is likely to affect sustainability in implementing TF-CBT. Barnafrid therefore aims to appoint regional coordinators (facilitators) to advance implementation in the child psychiatry and supervise sustainability and fidelity. The training programs and the implementation strategies in TF-CBT are part of the Barnafrid assignment to gather and disseminate knowledge about violence against children and evidenced-based trauma treatment.

3) Research on TF-CBT: Only a limited number of studies have been conducted on TF-CBT in Sweden, focusing on outcome trajectories, experiences of treatment and ongoing victimization and safety planning. The published studies come from the same study group (see supplement).

## Discussion

This study aimed to inform on the state of evidence-based care for traumatized children and adolescents in several European countries, with a particular focus on TF-CBT.

### Identified barriers

Several common barriers and successful D&I strategies were identified. Similar to Harvey and Gumport [19], we identified different levels of (modifiable) barriers to the further D&I of trauma-focused EBTs and of TF-CBT in particular, in Europe. Instead of a patient level, we identified a trauma-related level which highlighted the necessity to address barriers that were only related to trauma and could not be found in other mental health interventions. Factors such as trauma-related myths or lack of trauma sensitivity may have an impact not only on the patient but also on the therapist, organization and government levels. On the government level, the country narratives further highlighted the significant differences in European mental health services for trauma-informed care with their differing assessment tools and treatments being available in each country. Generally speaking, governments showed varying degrees of interest in supporting the implementation of EBTs (this is also reflected in the funding opportunities). The major differences in mental health systems may also reflect differing implementation strategies (e.g. development of specific training institutes or engagement strategies). The most frequently implemented treatments were EMDR and TF-CBT. TF-CBT is available in many different settings ranging from mental health clinics to schools. Differences in the previous training of therapists who learn TF-CBT further reflect the different pathways to qualification as a therapist in each country. A major treatment level barrier, that most trainers identified in the survey, was the lack of standardized criteria for participation in TF-CBT training and a standardized TF-CBT certification process that recently trained clinicians were bound to follow. Most European countries have their own training requirements, adapted to the different care and education systems, making it difficult to follow a unified model of implementation. A frequent exchange between countries on potential joint European certification process guidelines might further align these processes, and help countries which have just started dissemination of TF-CBT or any EBT by developing an effective program.

Trainers from three countries reported that training program were not required to be implemented by certified trainers but could be conducted by non-certified trainers or even clinicians who were not trained in TF-CBT. This may be due, in part, to the fact that in several countries there is no head organization restricting non-trained therapists (who may have simply read the manual) from offering trainings on TF-CBT (or other EBTs) to interested therapists.

Consequently, an official website with a list of certified international trainers (either per country or a general list for Europe), along with the creation of guidelines for a certification process (which could then be adapted to the specific country-related context) could protect the quality of the training programs.

Some others reported challenges related to the lack of translated materials and resources that were needed to improve TF-CBT dissemination and the quality of training programs. For example, several countries such as Italy do not have a translated version of the TF-CBT web-training, or in other countries outside of those included in the study, there might not be a manual translated to the local language. More unified criteria for training and further funding (e.g. for translations) are, therefore, needed to support the dissemination of the additional resources which may increase understanding and lead to the improved practice of the model.

### Successful D&I strategies

In terms of successful D&I strategies across countries, the results of the survey and country narratives identified vast numbers of trained clinicians in most countries, as well as the development of special training institutes or collaborations. Many countries also developed materials in their own language to overcome potential language barriers.

Another way forward in implementing the treatment in other countries could be to share funding models and experiences. This could facilitate the translation of materials, the development of training program and data collection for research purposes. One example of such an international effort is the project “TF-CBT Ukraine” in which international TF-CBT trainers, the developers of the treatment and local Ukrainian partners developed a training model for Ukrainian therapists. The large collaboration enabled the organization of international funding for the translation of therapy materials into Ukrainian and Russian and to translate the entire training program from English to Ukrainian [36].

In addition to a generally high density of research on TF-CBT conducted in Europe (see online supplement), we also identified several countries in which research on D&I of TF-CBT (Netherlands, Norway, Germany) was already being conducted. It is important to note that in almost all described TF-CBT D&I efforts in this manuscript, the treatment developers functioned as continuous consultants and made an essential contribution to the facilitation of the D&I of TF-CBT by training international trainers who could then go on implementing trainings and case consultations in their countries.

### Limitations

Firstly, the time period between the online survey and country narratives was rather long and several factors may have changed by the time the country narratives had been conducted. For example, there were already 20 trained clinicians in Italy at the time when the country narrative was written. Secondly, there could be a potential selection bias in online surveys and narratives as all trainers were invited to the survey, but only individual experts in the field were asked to write the country narrative. Additionally, the authors of the country narratives (all co-authors of this manuscript) are extremely well versed in the topic matter, but not very diverse (regarding e.g. minority backgrounds), conveying a potential for bias. Although all TF-CBT trainers are well-connected in their countries, it might be difficult for the single authors who wrote the country narratives to know about all initiatives and practices across a whole country. In future research it would be very interesting to add more perspectives on current D&I efforts, for example from clinicians who are or are not implementing the treatment. Thirdly, participants in the survey could also be the authors of the country narratives. Since the survey was implemented anonymously, we cannot exclude this potential source of bias. Fourthly, we neither assessed nor reported demographic information on the trainers as this might have made it easy to identify them. Fifthly, these results are not generalizable to other countries and mental health systems for children and adolescents in Europe as the systems are too heterogeneous. Lastly, the sample size was small which may limit the generalizability of the results.

## Conclusions and implications

This study showed that there are not only barriers but also great solutions for sustainably implementing and disseminating a trauma-focused treatment, which was originally developed in the USA, in the European context. The most central barriers of implementation and dissemination of TF-CBT were at a government and treatment level. Most of our participants expressed that European countries differ in terms of trauma related assessment tools and EBTs utilized to treat children affected by trauma. Therefore, future D&I efforts in Europe would benefit from the use of evidence-based engagement strategies to incorporate TF-CBT, the gold standard trauma treatment for children and adolescents who have experienced trauma, into the treatment policies and guidelines of each country (e.g. SAMHSA Model Program in the US). At a treatment level, we identified three main barriers: (1) lack of standardized criteria for participation in a TF-CBT training (as a therapist), (2) no requirement to conduct TF-CBT trainings by a certified trainer, and (3) lack of standardized TF-CBT certification process. Several solutions can be implemented to address each of these barriers. First, joint European efforts to create national guidelines with respect to the TF-CBT certification process for clinicians could be helpful. This would entail collaboration between countries to develop unified criteria for the eligibility of clinicians that can be enrolled. Subsequently, these criteria need to be applied to every specific country context. Second, by establishing guidelines for therapists’ certification, these should contain the requirement of having been trained by a certified TF-CBT trainer. Furthermore, European regional learning collaboratives may enhance the general popularity of the model and unify experts in the translation and dissemination of TF-CBT resources. In sum, we suggest to first consider the above mentioned government and treatment level barriers in order to overcome research to practice gaps – not only in European countries, but worldwide, which will then enable D&I efforts to focus on additional barriers at an organizational, therapist and trauma level. Additionally, an overarching factor across both levels of barriers was funding. Therefore, for the successful D&I of EBTs across European countries, we suggest sharing funding models and experiences (e.g., TF-CBT Ukraine), together with multi agency collaborations between training institutes, psychology networks and grant foundations to ensure additional resources.

PTSD symptom reductions after TF-CBT treatment in routine care in Norway [46] were similar to those of the Norwegian RCT [21] which is motivating for scholars to translate RCT protocols into routine clinical care even more. Hence, future research on D&I needs to focus on the dissemination of TF-CBT and other EBTs in other European countries such as Ukraine, with lessons learnt from the countries that have already implemented the model successfully. Policies around “gold standard” treatments by informing the public at large but also more specifically leaders in political circles and hospitals about the advantages of EBTs could facilitate this process at large. Moreover, additional research is needed to disentangle which aspects of the D&I processes are relevant in obtaining treatment outcomes.

## Supplementary Information

Supplementary Material 1.

Supplementary Material 2.

Supplementary Material 3.

## Data Availability

The data are available upon request from the authors.

## Abbreviations

CAP: Child and adolescent psychotherapists
CBITS: Cognitive Behavioural Intervention for Trauma in Schools
CBT: Cognitive behavioural therapy
D&I: Dissemination and implementation
EBTs: Evidence-based therapies
PTSD: Posttraumatic stress disorder
RCT: Randomized controlled trial
TF-CBT: Trauma-focused cognitive behavioural therapy
TTT: Train the trainer

## Footnotes

1 TF-CBT therapists are psychologists/psychotherapists or psychiatrists in the respective countries who have completed a TF-CBT training program run by a certified TF-CBT trainer.
