# Supplementary material for: The dissemination and implementation of trauma-focused cognitive behavioural therapy for children and adolescents in seven European countries
Source: BMC Health Serv Res. 2024 Oct 8;24:1202. doi: 10.1186/s12913-024-11689-3 (PMC11460130; doi:10.1186/s12913-024-11689-3)
Supplement: Supplementary file 1 — Supplementary Material 1. [file 12913_2024_11689_MOESM1_ESM.docx]

**Additional file 1: Questionnaire TF-CBT implementation Europe**

My TF-CBT persona

| Question | Answer Options (blank = open question) |
| --- | --- |
| In which country do you currently live and work? | *blank* |
| Please describe your TF-CBT experience. | |
| 1. TF-CBT as therapist/ clinician | - Yes, number of TF-CBT treatment cases: blank - no |
| 1. TF-CBT as certified trainer (TTT course) | - Yes - no |
| 1. Implement TF-CBT 2-3 day basic trainings | - Yes - no |
| 1. Implement TF-CBT case consultation/ supervision | - Yes - no |
| 1. As a researcher | - Yes - no |
| In which institutions/ settings do you personally deliver TF-CBT as part of your clinical work? | - Mental health clinics -inpatient setting - Mental health clinics - outpatient setting - General hospitals - Psychotherapy Training institutes - Schools - Child welfare programs - Juvenile justice system - Private Practice - Other: blank |
| In which institutions/ settings do you personally implement TF-CBT training? | - Mental health clinics -inpatient setting - Mental health clinics - outpatient setting - General hospitals - Psychotherapy Training institutes - Schools - Child welfare programs - Juvenile justice system - Private Practice - Other: blank |

Qualifications

1. TF-CBT Therapist in your respective country

| Question | Answer Options (blank = open question) |
| --- | --- |
| How many therapists/ clinicians were trained in TF-CBT in your country **altogether**? (approximate number) | *blank* |
| How many therapists/ clinicians are trained in TF-CBT in your country **each year**? (approximate number) | *blank* |
| How many therapists/ clinicians currently deliver TF-CBT in your country? (approximate number) | *blank* |
| Please describe the training requirements of a person who would participate in TF-CBT training as a therapist/ clinician. | - High School diploma/ A-level degree - Bachelor Degree in University studies (psychology, medicine, social studies, other) - Masters Degree in University studies (psychology, medicine, social studies, other) - Clinical training as part of a university degree (as part of Bachelor degree studies) - Clinical training as part of a university degree (as part of master degree studies) - Clinical training program after university degree (e.g. at institutes or schools for psychotherapy) - Part of vocational training/ After vocational training - Certain specializations (e.g. clinical social workers) - Other: *blank* |
| Do participants need a special degree in the following disciplines? | - Psychology - Clinical psychology - Psychiatry - Social work - Other: - No |
| Do therapists in your country need a licensure to be able to participate in TF-CBT training as a therapist/ clinician? | - No - Yes |
| If yes, please describe the licensure and requirements to obtain the licensure | *blank* |
| Is prior CBT experience/ training a requirement in order to become a TF-CBT therapist? | - Yes - Sometimes - No |
| If not, please state other theoretical backgrounds (e.g. psychodynamic, systemic) the therapists might have. | *blank* |
| How many years of experience in clinical work do therapists/ clinicians normally have before participation in TF-CBT training? (approximate range) | *blank* |
| What are necessary TF-CBT-related pre-conditions to be able to deliver TF-CBT as a therapist/ clinician in your country? | - TF-CBT web training (if available) - Read TF-CBT manual - Participate in 2-3 day basic training - Case consultation - Specific number of training cases under supervision - Other: *blank* |

TF-CBT trainers who completed the train-the-trainer program by the TF-CBT developers

| Question | Answer Options (blank = open question) |
| --- | --- |
| How many persons were trained as TF-CBT trainers by the developers in your respective country? | *blank* |
| How many TF-CBT trainings do the trainers in your country each offer every year? (approximate number) | *blank* |
| For how many TF-CBT therapists do the trainers in your country currently offer case consultation? (approximate number) | *Blank* |
| What **are currently** the necessary requirements for someone to be suggested for training as a TF-CBT trainer by the treatment developers? | *Blank* |
| What **should be** the necessary requirements for someone to be suggested for training as a TF-CBT trainer by the treatment developers? | *Blank* |

TF-CBT implementation in your mental health context

| Question | Answer Options (blank = open question) |
| --- | --- |
| In which institutions and settings is TF-CBT normally delivered by therapists/ clinicians? | - Mental health clinics inpatient setting - Mental health clinics outpatient setting - General Hospitals - Psychotherapy Training institutes - Schools - Child welfare programs - Juvenile justice system - Private Practice - Other: blank |
| Is TF-CBT delivered in in- or outpatient settings? | - Inpatient - Outpatient - both |
| Is TF-CBT training normally accompanied by case consultation? | - Yes - No |
| If not, what are the reasons? | *blank* |
| If yes, how many sessions of case consultation per therapist? | *blank* |
| Please briefly describe the content of the case consultations | *blank* |
| How many participants are normally in one case consultation? | *blank* |
| How many trainers are normally in one case consultation. | *blank* |
| If yes, how many training cases need to be completed with case consultation? | *blank* |
| Who offers TF-CBT trainings? | - TF-CBT therapist - TF-CBT trainer (certified by the developers) - Any clinician - Other: *blank* |
| Who offers TF-CBT case consultation? | - TF-CBT therapist - TF-CBT trainer (certified by the developers) - Any clinician - Other: *blank* |
| Is there a program for certification/ a license as TF-CBT therapist in your respective country? | *blank* |
| What do you think **should be** necessary requirements for getting licensed as a TF-CBT therapist? | *blank* |

TF-CBT dissemination

| Question | Answer Options (blank = open question) |
| --- | --- |
| What is the current dissemination in your country like? (e.g. several training institutes for clinicians, manual publication, scientific studies, certified trainers train others, development of toolbox or workbook in your language, training programs…) | - Develop training institutes which offer   TF-CBT training   - Building cooperations with institutes (e.g. mental health clinics) in order to implement TF-CBT - Development of evidence-based engagement strategy - Development of an implementation manual - Development of regional learning collaboratives for agencies/ institutions/ centers - Development of own “train-the-trainer” program - manual publication in respective language - publication of scientific studies onTF-CBT - Development of toolbox or workbook in your language - development of TF-CBT web training - others: *blank* |
| What do you think are barriers for dissemination of TF-CBT in your country? | *blank* |
| What do you think is necessary for further dissemination of TF-CBT in your respective country? | *blank* |
