# Supplementary material for: The dissemination and implementation of trauma-focused cognitive behavioural therapy for children and adolescents in seven European countries
Source: BMC Health Serv Res. 2024 Oct 8;24:1202. doi: 10.1186/s12913-024-11689-3 (PMC11460130; doi:10.1186/s12913-024-11689-3)
Supplement: Supplementary file 2 — Supplementary Material 2. [file 12913_2024_11689_MOESM2_ESM.docx]

**Additional file 2: Instructions for country narratives**

A) Please describe the regular mental health care for traumatized children/adolescents in your country. Please be sure to cover the following points: routine trauma screening in the mental health system; availability and access of trauma-focused treatments; the main trauma treatments used in your country (EMDR, psychodynamic, CBT, etc.); implementation of and attitude towards or obligation to provide special funding for evidence-based practices.

B) Please describe any research on TF-CBT and other trauma-focused treatments for children/adolescents in your country: How did TF-CBT initially arrive in your country? Please name or cite the TF-CBT research studies that have been completed. Please describe recent research on TF-CBT taking place in your country. Please describe recent research on other trauma-focused treatments for children and youth in your country.
