# Supplementary material for: The dissemination and implementation of trauma-focused cognitive behavioural therapy for children and adolescents in seven European countries
Source: BMC Health Serv Res. 2024 Oct 8;24:1202. doi: 10.1186/s12913-024-11689-3 (PMC11460130; doi:10.1186/s12913-024-11689-3)
Supplement: Supplementary file 3 — Supplementary Material 3. [file 12913_2024_11689_MOESM3_ESM.docx]

**Additional file 3: Overview TF-CBT research**

| **Theme (clinical trial, pilot study, mechanisms, adaptations, dissemination/ implementation (D&I))** | **Citations** | **Country** |
| --- | --- | --- |
| **Clinical trial** | Diehle, J., Opmeer, B.C., Mannarino, A., Boer, F., & Lindauer, R.J.L. (2014). Trauma-focused Cognitive Behavioral Therapy or Eye Movement Desensitization and Reprocessing - what works in children with posttraumatic stress symptoms? A randomized controlled trial. *European Journal of Child and Adolescent Psychiatry, 24*(2), 1-10.  Goldbeck, L., Muche, R., Sachser, C., Tutus, D., & Rosner, R. (2016). Effectiveness of Trauma-Focused Cognitive Behavioral Therapy for Children and Adolescents: A Randomized Controlled Trial in Eight German Mental Health Clinics. *Psychotherapy and Psychosomatics*, *85*(3), 159–170. <https://doi.org/10.1159/000442824>  Hultmann, O., Broberg, A. G., & Axberg, U. (2023). A randomized controlled study of trauma focused cognitive behavioural therapy compared to enhanced treatment as usual with patients in child mental health care traumatized from family violence. *Children and Youth Services Review, 144,* 106716.  Jensen, T. K., Holt, T., Ormhaug, S. M., Egeland, K., Granly, L., Hoaas, L., Stormyren, S., Indregaard, T., & Wentzel-Larsen, T. (2013). A randomized effectiveness study comparing trauma- focused cognitive behavioral therapy with therapy as usual for youth. *Journal of* *Clinical Child & Adolescent Psychology*. doi: 10.1080/15374416.2013.822307  Jensen, T. K., Holt, T., & Ormhaug, S. M. (2017). A Follow-up Study from a Multisite, Randomized Controlled Trial for Traumatized Children Receiving TF-CBT. *Journal of Abnormal Child Psychology, 45*(8), 1587-1597. doi:10.1007/s10802-017-0270-0  Tutus, D., Pfeiffer, E., Rosner, R., Sachser, C., & Goldbeck, L. (2017). Sustainability of Treatment Effects of Trauma-Focused Cognitive-Behavioral Therapy for Children and Adolescents. *Psychotherapy and psychosomatics*, *86*(6), 379-381. | The Netherlands  Germany  Sweden  Norway  Norway  Germany |
| **Mechanism:** change in cognitions, caregivers, alliance | Jensen, T. K., Holt, T., Ormhaug, S. M., Fjermestad, K., & Wentzel-Larsen, T. (2018). Change in post-traumatic cognitions mediates treatment effects for traumatized youth — A randomized controlled trial. *Journal of Counseling Psychology, 65*(2), 166-177. doi:10.1037/cou0000258  Birkeland, M. S., Holt, T., Ormhaug, S. M., & Jensen, T. K. (2020). Perceived social support and posttraumatic stress symptoms in children and youth in therapy: A parallel process latent growth curve model. *Behaviour Research and Therapy, 132*. doi:10.1016/j.brat.20 20.103655.  Holt, T., Cohen, J. A., Mannarino, A., & Jensen, T. K. (2014). Parental emotional response to children’s traumas. *Journal of Aggression, Maltreatment & Trauma,* 23(10), 1057-1071.  Holt, T., Jensen, T. K., & Wentzel-Larsen, T. (2014). The change and the mediating role of parental emotional reactions and depression in the treatment of traumatized youth: Results from a randomized controlled study. *Child and Adolescent Psychiatry and Mental Health*. doi: 10.1186/1753-2000-8-11  Loos, S., Tutus, D., Kilian, R., & Goldbeck, L. (2020). Do caregivers’ perspectives matter? Working alliances and treatment outcomes in trauma-focused cognitive behavioural therapy with children and adolescents. *European Journal of Psychotraumatology*, *11*(1), 1753939.  Ormhaug, S. M., Jensen, T. K., Wentzel-Larsen, T., & Shirk, S. R. (2014). The therapeutic alliance in treatment of traumatized youth: Relation to outcome in a randomized clinical trial. *Journal of Consulting and Clinical Psychology, 82*(1), 52-64.  Ormhaug, S. M., Shirk, S. R., & Wentzel-Larsen, T. (2015). Therapist and client perspectives on the alliance in the treatment of traumatized adolescents. *European Journal of Psychotraumatology, 6*. doi:10.3402/ejpt.v6.27705  Ovenstad, K. S., Jensen, T., & Ormhaug, S. M. (2021). Four perspectives on traumatized youth’s therapeutic alliance: Correspondence and outcome predictions. *Psychotherapy Research*. doi:10.1080/10503307.2021.2011983  Ovenstad, K. S., Ormhaug, S. M., Shirk, S. R., & Jensen, T. K. (2020). Therapists' behaviors and youth's therapeutic alliance during trauma-focused cognitive behavioral therapy. *Journal of Consulting and Clinical Psychology, 88*(4), 350-361. doi:10.1037/ccp0000465  Ovenstad, K. S., Ormhaug, S. M., & Jensen, T. K. (2022). The relationship between youth involvement, alliance and outcome in trauma-focused cognitive behavioral therapy. *Psychotherapy Research, ahead-of-print*(ahead-of-print), 1-12. doi:10.1080/10503307.2022.2123719  Pfeiffer, E., Sachser, C., de Haan, A., Tutus, D., & Goldbeck, L. (2017). Dysfunctional posttraumatic cognitions as a mediator of symptom reduction in Trauma-Focused Cognitive Behavioral Therapy with children and adolescents: Results of a randomized controlled trial. *Behaviour Research and Therapy*, *97*, 178–182. <https://doi.org/10.1016/j.brat.2017.08.001>  Tutus, D., Goldbeck, L., Pfeiffer, E., Sachser, C., & Plener, P. L. (2019). Parental dysfunctional posttraumatic cognitions in trauma-focused cognitive behavioral therapy for children and adolescents. *Psychological Trauma: Theory, Research, Practice, and Policy*, *11*(7), 722.  Tutus, D., Keller, F., Sachser, C., Pfeiffer, E., & Goldbeck, L. (2017). Change in parental depressive symptoms in trauma-focused cognitive-behavioral therapy: Results from a randomized controlled trial. *Journal of child and adolescent psychopharmacology*, *27*(2), 200-205.  Tutus, D., Pfeiffer, E., Plener, P. L., Rosner, R., Bernheim, D., & Sachser, C. (2021). The Change in Parental Symptoms and Dysfunctional Cognitions in the Course of Trauma-Focused Cognitive-Behavioral Therapy: Sustainability Until One-Year Post-Treatment. *Journal of child and adolescent psychopharmacology*, *31*(2), 129-136. | Norway  Norway  Norway  Norway  Germany  Norway  Norway  Norway  Norway  Norway  Germany  Germany  Germany  Germany |
| **Neurobiology (predictors and treatment effects)** | Zantvoord, J.B., Ensink, J.B.M., Kelder, op den R., Wessel, A.M.A., Lok, A., & Lindauer, R.J.L. (2019). Pretreatment cortisol predicts trauma-focused psychotherapy response in youth with (partial) posttraumatic stress disorder. *Psychoneuroendocrinology*, *109*, [104380]. https://doi.org/10.1016/j.psyneuen.2019.104380.  Zantvoord, J.B., Zhutovsky, P., Ensink, J.B.M., Kelder, R. op den, Wingen, G.A. van, & Lindauer, R.J.L. (2021). Trauma-focused psychotherapy response in youth with posttraumatic stress disorder is associated with changes in insula volume. *Journal of Psychiatric Research*, 132, 207-214. https://doi.org/10.1016/j.jpsychires.2020.10.03.  Zhutovsky, P., Zantvoord, J.B., Ensink, J.B.M., Kelder, R. op den, Lindauer, R.J.L., & van Wingen, G.A. (2021). Individual prediction of trauma-focused psychotherapy response in youth with posttraumatic stress disorder using resting-state functional connectivity*. NeuroImage: Clinical, 32*, [102898]. https://doi.org/10.1016/j.nicl.2021.10289. | The Netherlands  The Netherlands  The Netherlands |
| **Adaptation:** TF-CBT with refugee youth | Unterhitzenberger, J., Eberle-Sejari, R., Rassenhofer, M., Sukale, T., Rosner, R., & Goldbeck, L. (2015). Trauma-focused cognitive behavioral therapy with unaccompanied refugee minors: a case series. *BMC psychiatry*, *15*(1), 1-9.  Unterhitzenberger, J., & Rosner, R. (2016). Case report: manualized trauma-focused cognitive behavioral therapy with an unaccompanied refugee minor girl. *European Journal of Psychotraumatology*, *7*(1), 29246.  Unterhitzenberger, J., Wintersohl, S., Lang, M., König, J., & Rosner, R. (2019). Providing manualized individual trauma-focused CBT to unaccompanied refugee minors with uncertain residence status: a pilot study. *Child and Adolescent Psychiatry and Mental Health*, *13*(1), 1–10. | Germany  Germany  Germany |
| **D&I:** TF-CBT cultural adaptations | Unterhitzenberger, J., Haberstumpf, S., Rosner, R., & Pfeiffer, E. (2021). “Same Same or Adapted?” Therapists’ Feedback on the Implementation of Trauma-Focused Cognitive Behavioral Therapy With Unaccompanied Young Refugees. *Clinical Psychology in Europe*, *3*, 1-12. | Germany |
| **D&I:** TF-CBT impact of therapists | Pfeiffer, E., Ormhaug, S. M., Tutus, D., Holt, T., Rosner, R., Wentzel Larsen, T., & Jensen, T. K. (2020). Does the therapist matter? Therapist characteristics and their relation to outcome in trauma-focused cognitive behavioral therapy for children and adolescents. *European journal of Psychotraumatology*, *11*(1), 1776048. | Germany, Norway |
| **Adaptation:** group intervention based on TF-CBT | Pfeiffer, E., Sachser, C., Rohlmann, F., & Goldbeck, L. (2018). Effectiveness of a trauma-focused group intervention for young refugees: a randomized controlled trial. *Journal of Child Psychology and Psychiatry and Allied Disciplines*, *59*(11), 1171–1179. <https://doi.org/10.1111/jcpp.12908>  Pfeiffer, E., Sachser, C., Tutus, D., Fegert, J. M., & Plener, P. L. (2019). Trauma-focused group intervention for unaccompanied young refugees: “Mein Weg”—predictors of treatment outcomes and sustainability of treatment effects. *Child and adolescent psychiatry and mental health*, *13*(1), 1-10.  Pfeiffer, E., & Goldbeck, L. (2017). Evaluation of a trauma‐focused group intervention for unaccompanied young refugees: A pilot study. *Journal of Traumatic Stress*, *30*(5), 531-536. | Germany  Germany  Germany |
| **D&I:** Trials on TF-CBT dissemination | Rosner, R., Barke, A., Albrecht, B., Christiansen, H., Ebert, D. D., Lechner-Meichsner, F., Muche, R., Zarski, A. C., & Steil, R. (2020). BEST FOR CAN–bringing empirically supported treatments to children and adolescents after child abuse and neglect: study protocol. *European Journal of Psychotraumatology*, *11*(1), 1837531.  Rosner, R., Sachser, C., Hornfeck, F., Kilian, R., Kindler, H., Muche, R., Müller, L. R. F., Thielemann, J., Waldmann, T., Ziegenhain, U., Unterhitzenberger, J., & Pfeiffer, E. (2020). Improving mental health care for unaccompanied young refugees through a stepped-care approach versus usual care+: study protocol of a cluster randomized controlled hybrid effectiveness implementation trial. *Trials*, *21*(1), 1–13. https://doi.org/10.1186/s13063-020-04922-x | Germany  Germany |
| **D&I:** Implementation | Aminihajibashi, S., Skar, A. M. S., & Jensen, T. (2022). Professional wellbeing and turnover intention among child therapists: a comparison between therapists trained and untrained in Trauma-Focused Cognitive Behavioral Therapy. *BMC Health Services Research, 22*(1). doi:10.1186/s12913-022-08670- 3  Egeland, K. M., Skar, A. M. S., Endsjø, M., Laukvik, E. H., Bækkelund, H., Babaii, A., Granly, L. B., Granly, L., Husebø, G. K., Borge, R. H., Ehrhart, M. G., Sklar, M., Brown, C. H., & Aarons, G. A. (2019). Testing the leadership and organizational change for implementaion (LOCI) intervention in Norwegian mental health clinics: a stepped-wedge cluster randomized design study protocol. *Implementation Science, 14*(28). doi:10.1186/s13012-019-0873-7  Kasparik, B., Saupe, L. B., Mäkitalo, S., & Rosner, R. (2022). Online training for evidence-based child trauma treatment: evaluation of the German language TF-CBT-Web. *European Journal of Psychotraumatology, 13*(1), 2055890.  Pfeiffer, E., Beer, R., Birgersson, A., Cabrera, N., Cohen, J. A., Deblinger, E., ... & Klymchuk, V. (2023). Implementation of an evidence-based trauma-focused treatment for traumatised children and their families during the war in Ukraine: a project description. *European Journal of Psychotraumatology, 14(2),* 2207422. | Norway  Norway  Germany  Ukraine |
| **D&I:** Cost-effectiveness | Aas, E., Iversen, T., Holt, T., Ormhaug, S. M., & Jensen, T. K. (2019). Cost-Effectiveness Analysis of Trauma-Focused Cognitive Behavioral Therapy: A Randomized Control Trial among Norwegian Youth. *Journal of Clinical Child & Adolescent Psychology, 48*(1), S298-S311. doi:10.1080/15374416.2018.1463535  Aas, E., Silwal, S., Cyr, P. R., Holt, T., Ormhaug, S. M., & Jensen, T. K. (2020). Health-related quality of life (HRQoL) in children and adolescent with post-traumatic stress symptom: A comparison of 16D and condition-specific instruments. *Nordic Journal of Health Economics, 8*(1), 46-71. doi:10.5617/njhe.6929 | Norway  Norway |
| Other papers | Dittmann, I., & Jensen, T. K. (2014) Giving a voice to traumatized youth - Experiences with Trauma-focused cognitive behavioral therapy. *Child Abuse & Neglect.* 38. 1221–1230.  Jensen, T. K., Braathu, N., Birkeland, M. S., Ormhaug, S. M., & Skar, A.-M. S. (2022). Complex PTSD and treatment outcomes in TF-CBT for youth: a naturalistic study. *European Journal of Psychotraumatology, 13*(2), 2114630. doi:10.1080/20008066.2022.2114630  Knutsen, M., & Jensen, T. K. (2019). [Changes in the trauma narratives of youth receiving trauma-focused cognitive behavioral therapy in relation to posttraumatic stress symptoms](https://www.nkvts.no/vitenskapelig-artikkel/changes-in-the-trauma-narratives-of-youth-receiving-trauma-focused-cognitive-behavioral-therapy-in-relation-to-posttraumatic-stress-symptoms/). *Psychotherapy Research, 29*(1), 99. doi:10.1080/10503307.2017.1303208  Knutsen, M. L., Jensen, T. K., Sachser, C., Holt, T., & Goldbeck, L. (2019). Trajectories and Possible Predictors of Treatment Outcome for Youth Receiving Trauma-Focused Cognitive Behavioral Therapy. *Psychological Trauma*. doi:10.10 37/tra0000482  Onsjö, M., Strand, J., & Axberg, U. (2022). No child is an island–The life situation before and during treatment for children exposed to family violence. *Human systems, 2*(2), 64-81.  Onsjö, M., Strand, J., & Axberg, U. (2023). Children subjected to family violence: A retrospective study of experiences of trauma-focused treatment*. Clinical child psychology and psychiatry*, 13591045231169147.  Ormhaug, S. M., & Jensen, T. K. (2016). Investigating treatment characteristics and first-session relationship variables as predictors of dropout in the treatment of traumatized youth. *Psychotherapy Research*, 1-15. doi:10.1080/10503307.2016.1189617  Sachser, C., Pfeiffer, E., Tutus, D., & Rosner, R. (2019). Trauma-Focused Cognitive Behavioural Therapy (TF-CBT) for Children and Adolescents with PTSD and Complex PTSD: Secondary Analyses within a Randomized Controlled Trial. *European Journal of Psychotraumatology, 10(1*).  Skar, A.-M. S., Braathu, N., Jensen, T. K., & Ormhaug, S. M. (2022). Predictors of nonresponse and drop-out among children and adolescents receiving TF-CBT: investigation of client, therapist and implementation factors. *BMC Health Services Research, 22*, NA. Retrieved from<https://link-gale-com.ezproxy.uio.no/apps/doc/A720333697/AONE?u=oslo&sid=bookmark-AONE&xid=013ea9ae>  Unterhitzenberger, J., Sachser, C., & Rosner, R. (2020). Posttraumatic Stress Disorder and Childhood Traumatic Loss: A Secondary Analysis of Symptom Severity and Treatment Outcome. *Journal of Traumatic Stress, 33*(3), 208-217. doi:10.1002/jts.22499. | Norway  Norway  Norway  Norway  Sweden  Sweden  Norway  Germany  Norway  Germany |
